# Prevalence, Underlying Causes, and Preventability of Sepsis-Associated Mortality in US Acute Care Hospitals

**DOI:** 10.1001/jamanetworkopen.2018.7571

**Published:** 2019-02-15

**Authors:** Chanu Rhee, Travis M. Jones, Yasir Hamad, Anupam Pande, Jack Varon, Cara O’Brien, Deverick J. Anderson, David K. Warren, Raymund B. Dantes, Lauren Epstein, Michael Klompas

**Affiliations:** 1 Department of Medicine, Brigham and Women’s Hospital, Boston, Massachusetts; 2 Department of Population Medicine, Harvard Medical School, Harvard Pilgrim Health Care Institute, Boston, Massachusetts; 3 Duke Center for Antimicrobial Stewardship and Infection Prevention, Duke University School of Medicine, Durham, North Carolina; 4 Department of Medicine, Washington University School of Medicine at St Louis, St Louis, Missouri; 5 Department of Medicine, Duke University School of Medicine, Durham, North Carolina; 6 Division of Hospital Medicine, Emory University School of Medicine, Atlanta, Georgia; 7 Division of Healthcare Quality Promotion, Centers for Disease Control and Prevention, Atlanta, Georgia

## Abstract

**Importance:**

Sepsis is present in many hospitalizations that culminate in death. The contribution of sepsis to these deaths, and the extent to which they are preventable, is unknown.

**Objective:**

To estimate the prevalence, underlying causes, and preventability of sepsis-associated mortality in acute care hospitals.

**Design, Setting, and Participants:**

Cohort study in which a retrospective medical record review was conducted of 568 randomly selected adults admitted to 6 US academic and community hospitals from January 1, 2014, to December 31, 2015, who died in the hospital or were discharged to hospice and not readmitted. Medical records were reviewed from January 1, 2017, to March 31, 2018.

**Main Outcomes and Measures:**

Clinicians reviewed cases for sepsis during hospitalization using Sepsis-3 criteria, hospice-qualifying criteria on admission, immediate and underlying causes of death, and suboptimal sepsis-related care such as inappropriate or delayed antibiotics, inadequate source control, or other medical errors. The preventability of each sepsis-associated death was rated on a 6-point Likert scale.

**Results:**

The study cohort included 568 patients (289 [50.9%] men; mean [SD] age, 70.5 [16.1] years) who died in the hospital or were discharged to hospice. Sepsis was present in 300 hospitalizations (52.8%; 95% CI, 48.6%-57.0%) and was the immediate cause of death in 198 cases (34.9%; 95% CI, 30.9%-38.9%). The next most common immediate causes of death were progressive cancer (92 [16.2%]) and heart failure (39 [6.9%]). The most common underlying causes of death in patients with sepsis were solid cancer (63 of 300 [21.0%]), chronic heart disease (46 of 300 [15.3%]), hematologic cancer (31 of 300 [10.3%]), dementia (29 of 300 [9.7%]), and chronic lung disease (27 of 300 [9.0%]). Hospice-qualifying conditions were present on admission in 121 of 300 sepsis-associated deaths (40.3%; 95% CI 34.7%-46.1%), most commonly end-stage cancer. Suboptimal care, most commonly delays in antibiotics, was identified in 68 of 300 sepsis-associated deaths (22.7%). However, only 11 sepsis-associated deaths (3.7%) were judged definitely or moderately likely preventable; another 25 sepsis-associated deaths (8.3%) were considered possibly preventable.

**Conclusions and Relevance:**

In this cohort from 6 US hospitals, sepsis was the most common immediate cause of death. However, most underlying causes of death were related to severe chronic comorbidities and most sepsis-associated deaths were unlikely to be preventable through better hospital-based care. Further innovations in the prevention and care of underlying conditions may be necessary before a major reduction in sepsis-associated deaths can be achieved.

## Key Points

**Question:**

What is the prevalence of sepsis-associated mortality in US acute care hospitals and how preventable are these deaths?

**Findings:**

In this cohort study reviewing the medical records of 568 patients who were admitted to 6 hospitals and died in the hospital or were discharged to hospice and not readmitted, sepsis was present in 300 hospitalizations (52.8%) and directly caused death in 198 cases (34.9%). However, most underlying causes of death were related to severe chronic comorbidities and only 3.7% of sepsis-associated deaths were judged definitely or moderately preventable.

**Meaning:**

Sepsis is a leading cause of death in US hospitals, but most of these deaths are unlikely to be preventable through better hospital-based care.

## Teaser

This cohort study uses medical records to estimate the prevalence, underlying causes, and preventability of sepsis-associated mortality among adults in 6 US acute care hospitals.

## Introduction

Sepsis affects approximately 1.7 million adults in the United States each year and potentially contributes to more than 250 000 deaths.^1^ Various studies estimate that sepsis is present in 30% to 50% of hospitalizations that culminate in death.^1,2^ The high burden of sepsis and the perception that most sepsis-associated deaths are preventable with better care^3^ has catalyzed numerous sepsis performance improvement initiatives in hospitals around the world.

The extent to which sepsis-associated deaths in adults might be preventable, however, is unknown. Sepsis disproportionately affects patients who are elderly, have severe comorbidities, and have impaired functional status.^4,5,6,7^ Some of these patients may receive optimal, guideline-compliant care yet still die due to overwhelming sepsis or from their underlying disease. The prevalence and preventability of sepsis-associated deaths is difficult to discern from administrative data and death certificates because hospital discharge codes do not indicate whether sepsis caused death, and death certificates are often completed incorrectly.^8,9,10,11^

Quantifying the prevalence and preventability of deaths from sepsis has important implications for sepsis treatment initiatives and informing hospital resource allocation. We sought to characterize the prevalence, underlying causes, and preventability of sepsis-associated deaths in hospitalized adult patients using detailed medical record reviews in 6 US academic and community hospitals.

## Methods

### Study Design and Population

This multicenter retrospective cohort study included patients aged 18 years or older who were admitted from January 1, 2014, through December 31, 2015, to 3 tertiary referral centers (Brigham and Women’s Hospital, Boston, Massachusetts; Barnes-Jewish Hospital, St Louis, Missouri; and Duke University Hospital, Durham, North Carolina) and 3 community hospitals (Brigham and Women’s Faulkner Hospital, Boston, Massachusetts; Missouri Baptist Medical Center, St Louis, Missouri; and Duke Regional Hospital, Durham, North Carolina). We identified all patients who died in the hospital or emergency department during the study period and randomly selected 100 cases from each hospital for structured medical record reviews. Patients newly referred to hospice were also included as a surrogate for death, as hospice is an increasingly common end-of-life destination for patients with sepsis.^1,12,13^ We excluded patients who were already enrolled in hospice prior to admission unless the patient died in the hospital because our goal was to focus on patients for whom hospice indicated an acute transition to end-of-life care. We also excluded patients who were rehospitalized after discharge to hospice in order to focus on those who died shortly after discharge. Study approval was obtained from the institutional review boards at Harvard Pilgrim Health Care Institute, Partners HealthCare, Washington University School of Medicine, and Duke University Health System with a waiver of informed consent because this was a retrospective study of patients who died during hospitalization, making collection of consent not feasible. This study followed the Strengthening the Reporting of Observational Studies in Epidemiology (STROBE) reporting guideline for cohort studies.^14^

### Abstraction Process

Medical record reviews were performed by experienced clinicians (C.R., T.M.J., Y.H., A.P., J.V., and C.O.) using a standardized data abstraction tool in REDCap^15^ (eAppendix 1 in the Supplement). We reviewed patients’ discharge summaries, admission and progress notes, medications, laboratory and microbiology test results, radiology reports, and pathology and autopsy records (when available) to determine patients’ demographics, hospitalization characteristics, comorbidities, presence or absence of sepsis, and cause of death. Fixed categories of race/ethnicity, as reported by patients in the medical records of each hospital, were abstracted and reported to characterize the generalizability of the study cohort.

We identified patients with end-stage comorbidities using the hospice eligibility criteria set by the Centers for Medicare & Medicaid Services (eTable 1 in the Supplement).^16^ The immediate and underlying causes of death were assigned using guidelines for completing death certificates from the Centers for Disease Control and Prevention.^17^ Specifically, the immediate cause of death was defined as the final disease, injury, or complication causing death, while the underlying cause of death was defined as the disease or injury that initiated the chain of events that led directly or inevitably to death. For patients who were discharged to hospice, the immediate cause of death was defined as the disease or injury that triggered the decompensation leading to a shift in the goals of care and transition to hospice.

Sepsis was defined as infection with a concurrent rise in Sequential Organ Failure Assessment Score^18^ by 2 or more points from the preinfection baseline, as per Sepsis-3 criteria.^19^ Sepsis cases were further classified as possible, probable, or definite based on the probability of infection and presence of other causes of organ dysfunction (eAppendix 2 in the Supplement). Hospital-onset sepsis was defined as sepsis arising from an infection acquired more than 48 hours after admission. Sepsis arising 48 hours or less from admission was defined as present on admission.

### Preventability Assessments and Reviewer Training

Reviewers were instructed to identify suboptimal aspects of sepsis care, including delays of more than 3 hours in the administration of broad-spectrum antibiotics from the onset of sepsis-associated organ dysfunction, inappropriate initial empirical antibiotic therapy relative to final culture results, delayed or inadequate fluid resuscitation, and delays in source control. Reviewers also identified procedure-related complications, adverse reactions to medications or errors in administration of medications, in-hospital falls, venous thromboembolism associated with inadequate prophylaxis, and hospital-acquired infections.

Reviewers made an overall assessment of the preventability of each death, taking into account patients’ comorbidities and functional status, severity of illness at sepsis onset, concurrent acute illnesses, goals of care, and quality of care using a Likert scale adapted from prior work on preventability.^20,21^ The Likert scale ranged from 1 to 6 (where 1 indicated definitely preventable; 2, moderately likely to be preventable; 3, potentially preventable under the best circumstances and optimal clinical care; 4, unlikely to be preventable even though some circumstances and some aspects of clinical care may not have been optimal; 5, moderately likely not to be preventable; and 6, definitely not preventable owing to rapidly fatal illness present on admission and/or goals of care on admission that precluded aggressive care). In assessing optimal clinical care, we instructed reviewers to consider best practices as defined by the Surviving Sepsis Campaign guidelines^22^ and to assume that all practices recommended in the guidelines could be provided even if, in reality, some services were not reliably available in their site. For example, if a patient with sepsis had a delay in source control owing to an unavailable surgeon or interventional radiologist, we instructed reviewers to consider this a preventable death. Furthermore, we instructed reviewers to consider reasonable clinical judgement based on knowledge that would have been available to the treating clinician in real time. For example, if a patient died from an unusual organism found only after intensive diagnostic efforts, that death would not be considered preventable so long as extensive diagnostic efforts were undertaken and reasonable care would not entail empirical treatment of that pathogen.

Each of the 3 sites used 2 reviewers trained by one of us (C.R.) to standardize the abstraction process. The first 30 records at each site were reviewed independently by the 2 reviewers, and the Krippendorff α coefficient^23^ was calculated to determine interrater reliability for the determination of whether sepsis was the cause of death, and the overall preventability rating (as an ordinal classification). Reviewers then discussed all discrepant classifications to find consensus and promote a uniform approach to all classifications. If the Krippendorff α coefficient was less than 0.6 for either sepsis classification or preventability of death, reviewers undertook an additional 15 medical record reviews and repeated the standardization process. All medical record reviews were conducted between January 1, 2017, and March 31, 2018.

### Statistical Analysis

Descriptive analyses were used to calculate the prevalence of sepsis in hospital deaths, comorbidity burden, prevalence and types of medical errors in deaths associated with sepsis, and overall preventability of deaths associated with sepsis. We calculated 95% CIs for the prevalence of sepsis and end-stage comorbidities and overall preventability using binomial distributions. The primary analysis included all cases of sepsis, but we performed a sensitivity analysis limited to definite and probable cases of sepsis. All *P* values were from 2-sided tests and results were deemed statistically significant at *P* < .05. Analyses were conducted using SAS, version 9.3 (SAS Institute Inc) and an online software package to calculate Krippendorff α.^24^

## Results

### Study Cohort and Patient Characteristics

The study cohort included 600 patients who died in the hospital or were discharged to hospice. We excluded 23 patients who were already receiving hospice care prior to admission (and did not die in the hospital) and an additional 9 patients who were discharged to hospice but were subsequently rehospitalized. Of the 568 patients included in the analysis, mean age (SD) was 70.5 (16.1) years, 289 (50.9%) were male, 395 (69.5%) died in the hospital, and 173 (30.5%) were discharged to hospice (Table 1). Of the 173 patients discharged to hospice, 59 (34.1%) died within 7 days of discharge, while 65 (37.6%) died after 7 days; dates of death could not be ascertained in the remaining 49 patients discharged to hospice (28.3%).

**Table 1.** Characteristics of Patients Who Died In the Hospital or Were Discharged to Hospice

Characteristic	Patients, No. (%)		*P* Value
	Sepsis Present (n = 300)	Sepsis Absent (n = 268)	
Age, mean (SD), y	70.8 (15.9)	70.3 (16.3)	.78
Male sex	155 (51.7)	134 (50.0)	.69
Race/ethnicity			
White	226 (75.3)	209 (78.0)	.71
Black	54 (18.0)	45 (16.8)	
Hispanic	10 (3.3)	5 (1.9)	
Other	10 (3.3)	9 (3.4)	
Preadmission location			
Home	222 (74.0)	236 (88.1)	<.001
Facility	78 (26.0)	32 (11.9)	
Hospital type			
Academic	146 (48.7)	135 (50.4)	.69
Community	154 (51.3)	133 (49.6)	
Admitting service			
Medical	257 (85.7)	207 (77.2)	.004
Surgical	31 (10.3)	28 (10.5)	
Other	12 (4.0)	33 (12.3)	
Type of admission			
Emergency	288 (96.0)	262 (97.8)	.11
Elective	12 (4.0)	6 (2.2)	
DNR or DNI on admission	67 (22.3)	63 (23.5)	.74
Required ICU admission	210 (70.0)	104 (38.8)	<.001
Unit location at death			
ICU	151/243 (62.1)	61/152 (40.1)	<.001
Non-ICU ward	91/243 (37.5)	65/152 (42.8)	
Emergency department	1/243 (0.4)	18/152 (11.8)	
Comorbidities			
Solid cancer	86 (28.7)	108 (40.3)	.004
Hematologic cancer	31 (10.3)	18 (6.7)	.13
Dementia	46 (15.3)	25 (9.3)	.03
Heart failure	73 (24.3)	55 (20.5)	.28
Liver disease	19 (6.3)	13 (4.9)	.45
Chronic lung disease	71 (23.7)	51 (19.0)	.18
Chronic renal disease	75 (25.0)	50 (18.7)	.07
Prior stroke	45 (15.0)	24 (9.0)	.03
Coronary disease	92 (30.7)	72 (26.9)	.32
Diabetes	102 (34.0)	77 (28.7)	.18
Substance abuse	14 (4.7)	14 (5.2)	.76
Hypertension	191 (63.7)	165 (61.6)	.61
Atrial fibrillation	74 (24.7)	70 (26.1)	.69
Hospitalization within prior year	185 (61.7)	144 (53.7)	.06
Hospitalization within prior 60 d	125 (41.7)	101 (37.7)	.33
Hospital LOS, median (IQR), d	9 (5-17)	5 (3-8.5)	<.001
ICU LOS, median (IQR), d	5 (2-10)	3 (1-6)	<.001
Death	243 (81.0)	152 (56.7)	<.001
Hospice	57 (19.0)	116 (43.3)	<.001

Abbreviations: DNI, do not intubate, DNR, do not resuscitate, ICU, intensive care unit, IQR, interquartile range; LOS, length of stay.

On medical record review, sepsis (possible, probable, or definite) was present in 300 (52.8%; 95% CI, 48.6%-57.0%) terminal hospitalizations. Sepsis was present on admission in 221 of these 300 cases (73.7%) and acquired in the hospital in the remaining 79 cases (26.3%). Patients who died with and without sepsis were similar with respect to age, sex, race/ethnicity, type of admission, do-not-resuscitate or do-not-intubate status on admission, hospitalizations within the prior year, and distribution of most comorbidities except solid cancers (less common in deaths from sepsis) (Table 1). However, patients with sepsis had higher rates of admission from acute rehabilitation or long-term care facilities vs home, admission to medical vs surgical or other services, admission to the intensive care unit, and death in the hospital as opposed to hospice.

End-stage comorbidities (defined by hospice criteria) were present on admission in 121 of the 300 patients with sepsis who died (40.3%; 95% CI, 34.7%-46.1%) (eTable 2 in the Supplement). The most common comorbidities were metastatic or progressive solid cancer (60 [20.0%]), refractory hematologic cancer (16 [5.3%]), severe debilitating dementia (15 [5.0%]), severe debilitating stroke (12 [4.0%]), or severe chronic lung disease (12 [4.0%]). Patients without sepsis who died were more likely to meet hospice criteria compared with patients with sepsis (135 of 268 [50.4%] vs 121 of 300 [40.3%]; *P* = .02), with a similar distribution of conditions except for solid cancer (less common in patients with sepsis) and prior stroke (more common in patients with sepsis).

Compared with patients with sepsis who died in academic hospitals, patients with sepsis who died in community hospitals were older; more likely to reside in facilities prior to admission; less likely to have cancer; and more likely to be admitted to medical services, to have do-not-resuscitate or do-not-intubate status on admission, and to die in non–intensive care unit locations (eTable 3 in the Supplement).

### Causes of Death

**Figure 1.**
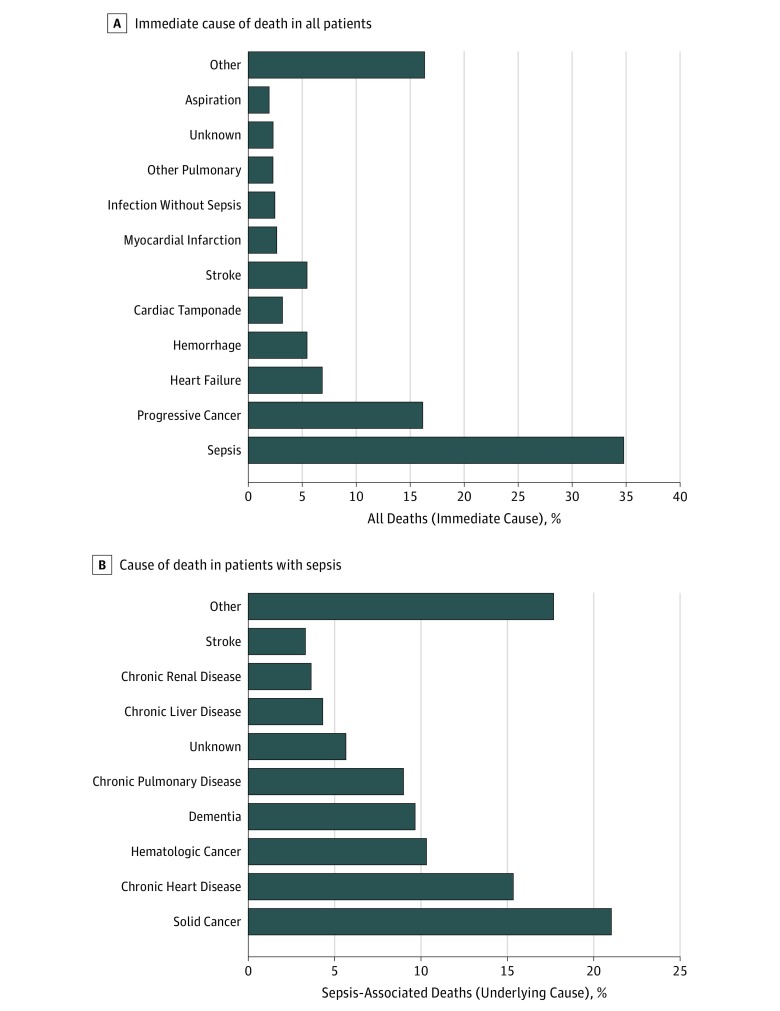
Distribution of Causes of Death A, Immediate cause of death among all patients (with and without sepsis). B, Underlying cause of death in patients with sepsis. The cohort included 568 patients who died in the hospital or were discharged to hospice, of whom 300 had sepsis at some point during hospitalization. Per Centers for Disease Control and Prevention guidelines, the immediate cause of death was defined as the final disease, injury, or complication causing death, while the underlying cause of death was defined as the disease or injury that initiated the chain of events that led directly or inevitably to death. For patients discharged to hospice, the immediate cause of death was considered to be the disease or injury that triggered the decompensation leading to a shift in goals of care and transition to hospice. Among the patients with sepsis as the immediate cause of death, 100 of 198 deaths (50.5%) were from pneumonia, 38 of 198 (19.2%) from intra-abdominal infections, 25 of 198 (12.6%) from endovascular infections, 19 of 198 (9.6%) from urinary infections, and 11 of 198 (5.6%) from unknown infectious source.

The distribution of immediate causes of death in the cohort is summarized in Figure 1A. Sepsis was the immediate cause of death in 198 patients (34.9%; 95% CI, 30.9%-38.9%). Sepsis was present during hospitalization in another 102 patients (18.0%; 95% CI, 14.9%-21.4%) but resolved prior to death. Nonetheless, clinician reviewers still thought the sepsis episode contributed to death in 44 of these 102 patients (43.1%; 95% CI, 33.4%-53.3%). The most common infectious source of sepsis among patients in whom sepsis was the immediate cause of death was pneumonia (100 of 198 [50.5%]), followed by intra-abdominal infections (38 of 198 [19.2%]) and endovascular infections (25 of 198 [12.6%]). After sepsis, the most common immediate causes of death were progressive cancer (92 of 568 [16.2%]) and heart failure (39 of 568 [6.9%]).

The most common underlying causes of death in patients with sepsis were solid cancer (63 of 300 [21.0%]), chronic heart disease (46 of 300 [15.3%]), hematologic cancer (31 of 300 [10.3%]), dementia (29 of 300 [9.7%]), and chronic lung disease (27 of 300 [9.0%]) (Figure 1B). Of the 243 of 300 patients (81.0%) with sepsis who died in the hospital, 151 (62.1%) died in the intensive care unit, 91 (37.5%) died on an inpatient ward, and 1 died in the emergency department (0.4%). When limiting the analysis to probable or definite sepsis (and not possible sepsis), sepsis was the immediate cause of death in 181 patients (31.9%; 95% CI, 28.1%-35.9%) and present during hospitalization without immediately contributing to death in another 43 patients (7.6%; 95% CI, 5.5%-10.1%).

### Preventability of Sepsis-Associated Deaths

**Figure 2.**
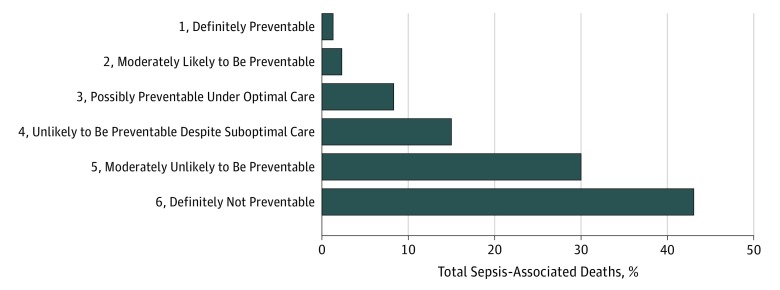
Distribution of Preventability Ratings for Patients With Sepsis Who Died The cohort included 300 patients with sepsis at some point during hospitalization who died or were discharged to hospice and not readmitted. Preventability assessments focused only on care received in the hospital and took into account patients’ comorbidities and functional status, severity of illness at sepsis onset, concurrent acute illnesses, goals of care, and quality of care (including any delays or errors in sepsis management). Preventability ratings: 1 indicates definitely preventable; 2, moderately likely to be preventable; 3, potentially preventable under the best circumstances and optimal clinical care; 4, unlikely to be preventable even though some circumstances and clinical care may not have been optimal; 5, moderately likely not to be preventable; and 6, definitely not preventable owing to rapidly fatal illness present on admission and/or goals of care on admission that precluded aggressive care.

Overall, 264 of the 300 deaths from sepsis (88.0%; 95% CI, 83.8%-91.5%) were considered unpreventable (4-6 rating on the Likert scale); only 36 deaths (12.0%; 95% CI, 8.6%-16.2%) were potentially preventable (11 [3.7%] definitely or moderately likely preventable and 25 [8.3%] possibly preventable) (Figure 2). There were no identifiable suboptimal aspects of care in 232 sepsis-associated deaths (77.3%). Of the 68 cases (22.7%) with suboptimal care, the most common problems were delay in antibiotics (33 [48.5%]), delay in source control (19 [27.9%]), and inappropriate initial empirical antibiotic therapy relative to final culture results (16 [23.5%]). Among these 68 cases, 32 deaths (47.1%) were judged to be definitely, moderately likely, or possibly preventable. Generally, the nonpreventable sepsis-associated deaths occurred in patients with major underlying comorbidities, severe acute concurrent illnesses, and/or florid sepsis that progressed through optimal care. A representative sample of potentially preventable and nonpreventable deaths among patients with sepsis is shown in Table 2.

**Table 2.** Representative Sample of Potentially Preventable vs Nonpreventable Sepsis-Associated Deaths

Case Summary	Underlying Cause of Death	Reasons Why Death Was Preventable or Nonpreventable
**Definitely (1) or Moderately Likely (2) to Be Preventable**^a^		
Elderly patient in relatively good health admitted for hyponatremia secondary to syndrome of inappropriate antidiuretic hormone secretion. On hospital day 4, patient developed a fever and then became confused. Antibiotics not started until blood cultures grew gram-positive cocci (later identified as methicillin-sensitive *Staphylococcus aureus*). Patient was noted then to have erythema and purulence at site of prior peripheral intravenous catheter. Infection thought to be secondary to catheter infiltration and septic thrombophlebitis. Despite operative thrombus excision, patient experienced septic shock, respiratory failure, and neurologic dysfunction secondary to cerebral septic emboli. Patient was transitioned to comfort measures and died.	Infection of peripheral venous catheter site with septic thrombophlebitis	Hospital-acquired vascular infection, delay in antibiotics (only started when blood cultures turned positive; could have been started earlier based on fevers, confusion, and infected catheter site)
Elderly patient with severe chronic obstructive pulmonary disease and atrial fibrillation was admitted for elective partial lobectomy that was complicated by major bleeding that contributed to persistent respiratory failure. Patient was then extubated but had hypoxia for several days afterward (not treated with antibiotics despite sputum cultures positive for *Escherichia coli* and *Serratia marcescens* and infiltrates detected on chest radiograph). Patient was then reintubated for pneumonia that progressed to septic shock; ultimately, patient was transitioned to comfort measures and died.	Chronic obstructive pulmonary disease	Procedural complication (bleeding); delay in antibiotics for pneumonia
Elderly patient with chronic obstructive pulmonary disease, diabetes, coronary artery disease, and gastric cancer in remission after gastrectomy presented with constipation and high-grade small-bowel obstruction. Afebrile but hypotensive with 24% bands and elevated lactate. No antibiotics given. Admitted to surgical ward with conservative management. Next morning, patient had worsening abdominal pain, bandemia, lactic acidosis, and oliguria. Taken to operating room in late afternoon. No antibiotics given until patient was in the operating room. Found to have necrotic bowel that was resected. Postoperatively, patient had septic shock physiology and was transitioned to comfort measures and died. No evidence of recurrent gastric cancer on autopsy.	Gastric cancer (remote, but led to surgery that led to bowel obstruction)	Delay in antibiotics and source control (earlier operative management prior to bowel infarction could have prevented sepsis)
**Potentially Preventable (3)**^a^		
Middle-aged patient with lymphoma and history of hematopoietic stem cell transplant admitted with *Clostridium difficile* diarrhea and dyspnea of unclear cause, thought to be graft-vs-host disease. No antibiotics given. Respiratory distress worsened. Patient then received a diagnosis of *Pneumocystis jiroveci* pneumonia based on findings of chest computed tomography and elevated beta-d-glucan but was not treated with trimethoprim-sulfamethoxazole until hospital day 4. Died from respiratory failure and sepsis.	Lymphoma	Delay in appropriate antibiotics (no anti-*Pneumocystis* therapy); rated potentially preventable instead of moderate or definite because of atypical pathogen that many clinicians do not typically cover in empirical regimens for community-acquired or health care–acquired pneumonia
Elderly patient with coronary artery disease, aortic valve replacement, heart failure, atrial fibrillation, diabetes, chronic kidney disease, and peripheral vascular disease was admitted with hypotension, renal failure, leukocytosis, and altered mental status, initially attributed solely to gastrointestinal bleeding. On hospital day 2, patient developed a fever and was treated with broad-spectrum antibiotics. Patient was found to have methicillin-resistant *S aureus* bacteremia with polyarticular septic arthritis and likely endocarditis. Patient eventually died from sepsis.	Valvular heart disease	Delay in sepsis recognition and antibiotics (no antibiotics for >24 h after presenting with signs and symptoms of sepsis, initially attributed to gastrointestinal bleeding); rated potentially preventable instead of moderate or definite because sepsis not clearly obvious on admission
Elderly patient with chronic obstructive pulmonary disease, a history of perforated diverticulitis requiring colon resection and complicated by enterocutaneous fistula, dependent on total parenteral nutrition, and with line-associated deep vein thrombosis (taking anticoagulant) was admitted with pneumonia and intra-abdominal abscess secondary to colonic leak. Patient received antibiotics, but no drainage was performed for 2 d while waiting for international normalized ratio to normalize. Patient developed altered mental status, respiratory distress, and then septic shock and died.	Diverticular disease	Delay in source control (no drainage of intra-abdominal abscess for 2 d); rated potentially preventable instead of moderate or definite because of reasonable concern for coagulopathy and procedure risk
**Unlikely to Be Preventable (Even Though Some Circumstances and Clinical Care May Not Have Been Optimal) (4)**^a^		
Elderly patient with no medical history presented with 2 wk of abdominal pain and change in stool caliber. White blood cell count was 29 000 cells/μL on admission. Abdominal computed tomography scan showed large obstructing colonic tumor with external invasion and contained perforation, with metastatic disease and peritoneal carcinomatosis. Patient received fluids and antibiotics and was admitted to surgical service with plan for operative intervention next morning. Overnight, patient developed hypotension, and intra-abdominal free air was detected on chest radiograph. Patient was taken emergently for surgery and found to have diffuse stool spillage in abdomen. Patient developed septic shock and multiorgan failure postoperatively. Family decided on comfort measures, and patient died.	Colon cancer	Under optimal circumstances, patient would have gone to surgery immediately on presentation, prior to perforation. However, decision to perform surgery in morning was not unreasonable at the time, and the prognosis was poor given the extent of the patient’s cancer
**Moderately (5) or Definitely (6) Unlikely to Be Preventable**^a^		
Middle-aged patient with alcohol abuse and smoking history presented with back pain, leg weakness, incontinence, hemoptysis, and falls at home. Severe hypoxemia on arrival requiring immediate intubation. Subsequent hypotension requiring vasopressors. Chest radiograph showed large right-sided pleural effusion with underlying lung mass and likely liver metastases. Chest tube placed with frank pus. Antibiotics immediately administered. Bronchoscopy showed large mass obstructing right mainstem bronchus. Patient experienced cardiac arrest (pulseless electrical activity) shortly after admission, was resuscitated, required 3 vasopressors, and had multiorgan failure. Patient was transitioned to comfort care and died.	Lung cancer (newly diagnosed)	Severely ill on arrival to hospital and underlying metastatic lung cancer causing bronchus obstruction; unlikely to have survived under any circumstances
Elderly patient with refractory acute myelogenous leukemia (treated with multiple rounds of chemotherapy) presented with fever, cough, hypotension, and multifocal pneumonia detected on chest radiograph. Despite timely broad-spectrum antibiotics, patient developed worsening delirium and multiorgan failure, with 90% blasts on peripheral smear. Palliative hydroxyurea was initiated, but goals of care changed to comfort measures. Patient was discharged to hospice where he died shortly after.	Acute myelogenous leukemia	Had sepsis from pneumonia on arrival but main underlying problem was progressive, incurable leukemia

SI conversion factor: To convert white blood cell count to ×10^9^/L, multiply by 0.001.

^a^ The numbers 1 through 6 in each preventability category correspond to the Likert scale used by clinician reviewers (1 indicates definitely preventable; 2, moderately likely to be preventable; 3, potentially preventable under the best circumstances and optimal clinical care; 4, unlikely to be preventable even though some circumstances and clinical care may not have been optimal; 5, moderately likely not to be preventable; and 6, definitely not preventable owing to rapidly fatal illness present on admission and/or goals of care on admission that precluded aggressive care).

A total of 42 major errors were identified in the 36 sepsis-associated deaths that were potentially preventable (Table 3). Most of the errors were related to delays in recognition and treatment of sepsis (n = 16), inappropriate antibiotic therapy administered after recognition of sepsis (n = 10), or delays in source control (n = 7). Two patients had potentially preventable hospital-acquired infections, while 3 had procedural complications (ie, bleeding and ischemia) and 3 had medication-related adverse events (ie, bleeding from excessive oral anticoagulation) that triggered a cascade of events leading to sepsis and death. One patient was inadequately monitored on a hospital ward after admission and had delayed recognition of an unstable arrhythmia. Of the 36 potentially preventable deaths, only 1 patient met criteria for hospice on admission (end-stage liver disease). This patient’s death was still considered possibly preventable, as he did not receive gram-negative antibiotic coverage for pneumonia caused by *Escherichia coli*.

**Table 3.** Summary of Major Errors Contributing to Potentially Preventable Sepsis-Associated Deaths and Specific Underlying Infections or Complications^a^

Major Error Category	Specific Infection or Complication
Delay in recognition of infection or sepsis, leading to delay in antibiotics or source control (n = 9)	Empyema (n = 1); enterococcal bacteremia (n = 1); necrotic bowel (n = 1); *Pseudomonas aeruginosa* pneumonia (n = 1); *Staphylococcus aureus* bacteremia (n = 4); *Staphylococcus lugdunensis* bacteremia (n = 1)
Infection or sepsis recognized but delay in antibiotics (n = 7)	Intra-abdominal infection (n = 1); necrotic bowel (n = 2); pneumonia, unknown pathogen (n = 2); *P aeruginosa* bacteremia (n = 1); sepsis of unclear source (n = 1)
Infection or sepsis recognized, timely antibiotics administered but inappropriate choice (n = 10)	*Bacteroides fragilis* bacteremia (no anaerobic coverage) (n = 1); *Candida albicans* bloodstream infection (no antifungal) (n = 1); *Escherichia coli* pneumonia (no gram-negative coverage) (n = 1); extended-spectrum beta-lactamase–producing *E coli* bacteremia (no carbapenem) (n = 1); *Enterococcus faecalis* bacteremia (no enterococcal coverage) (n = 1); methicillin-resistant *S aureus* bacteremia (no vancomycin) (n = 1); *Mycoplasma pneumoniae* encephalitis (no atypical coverage) (n = 1); *Pneumocystis jiroveci* pneumonia (no pneumocystis coverage) (n = 1); *P aeruginosa* pneumonia (no pseudomonal coverage) (n = 1); septic shock of unclear source (no gram-negative coverage, only vancomycin + azithromycin)
Infection or sepsis recognized but delay in source control (n = 7)	Chest tube for empyema (n = 1); percutaneous drainage of gangrenous gallbladder (n = 1); percutaneous drainage of intra-abdominal abscess (n = 1); removal of infected central line with *S aureus* bacteremia (n = 1); surgery for necrotic bowel (n = 3)
Potentially preventable hospital-acquired infection (n = 2)	Central line–associated bloodstream infection with *C albicans* (n = 1); peripheral intravenous catheter–associated septic thrombophlebitis with *S aureus* bacteremia (n = 1)
Procedural complication (n = 3)	Major bleeding after elective thoracic surgery (n = 1); major bleeding after paracentesis (n = 1); cardiac ischemia and myocardial infarction after elective arrhythmia ablation (n = 1)
Medication adverse event (n = 3)	Major bleeding from excessive oral anticoagulation (n = 2); chemotherapy adverse event (n = 1)
Other (n = 1)	Inadequate patient monitoring leading to delayed recognition of unstable arrhythmia (n = 1)

^a^ There were 36 potentially preventable sepsis-associated deaths in the cohort. The total number of errors in the table (n = 42) exceeds 36 because several patients experienced multiple major errors.

The preventability ratings were generally similar for sepsis-associated deaths in academic vs community hospitals and for patients with sepsis present on admission vs sepsis acquired in the hospital (eTable 4 and eTable 5 in the Supplement). The preventability ratings in the subset of patients whose immediate cause of death was sepsis was also similar to those with sepsis present at any time during hospitalization: 20 of 198 deaths (10.1%) were deemed possibly preventable and 10 (5.1%) were considered definitely or moderately preventable (eTable 6 in the Supplement).

### Interrater Reliability for Sepsis and Preventability Classifications

At the 3 sites, the initial Krippendorff α values for classifying sepsis as a cause of death were 0.85 (site 1), 0.32 (site 2), and 0.93 (site 3). The initial Krippendorff α values for classifying preventability of death were 0.60 (site 1), 0.52 (site 2), and 0.34 (site 3). After reconciling discrepant cases, retraining, and reviewing a second set of 15 overlapped cases, the Krippendorff α increased to 1.0 for classifying sepsis and 0.66 for preventability at site 2. At site 3, the Krippendorff α increased to 1.0 for classifying sepsis and 0.53 for preventability.

## Discussion

We found that sepsis was present in more than 50% of adult hospitalizations ending in death or discharge to hospice. In two-thirds of these cases, sepsis was the immediate cause of death. Patients who died with sepsis tended to be older adults with multiple comorbidities and recent hospitalizations, and underlying causes of death were mostly associated with severe chronic comorbidities. Approximately 40% of patients with sepsis who died met hospice-qualifying criteria on admission, most commonly terminal cancer. One in 8 sepsis-associated deaths were judged potentially preventable with better hospital-based care, including 1.3% that were considered definitely preventable, 2.3% considered moderately preventable, and 8.3% considered possibly preventable. Suboptimal sepsis care, such as delays in antibiotic administration or source control, were identified in 22.7% of patients with sepsis who died, but death was still thought to be unpreventable in more than half of those patients.

Our estimate of the prevalence of sepsis in hospital deaths is similar to prior analyses of large administrative and clinical databases.^1,2^ In contrast, studies based on death certificate data estimate that only 6% of deaths in the United States are associated with sepsis.^8,25^ Some of this discrepancy is owing to death certificates capturing deaths that occur outside the hospital, as national data from 2014 indicate that only 37% of deaths occur in the hospital.^26^ However, if half of hospital deaths are associated with sepsis, this finding still suggests that death certifications are inaccurate and incomplete with respect to coding for sepsis.^8,9,10,11^ Sepsis may be particularly susceptible to undercoding the cause of death because some clinicians may document infection alone, rather than sepsis, as the cause.^8^

Although the burden of sepsis-associated mortality is high, our study indicates that most of these deaths may not be preventable through better hospital-based care. Reviewers’ judgements of nonpreventability centered mainly on incurable underlying diseases as well as severe illnesses that were either treated appropriately yet progressed or were thought unlikely to have been affected by suboptimal aspects to care. Previous studies also found that most in-hospital deaths are probably not preventable, even when medical errors occur or the quality of medical care is suboptimal.^27,28^ Our findings extend the results of these prior investigations to encompass sepsis. In our cohort, metastatic or progressive cancer was the leading underlying cause of death in patients who died with sepsis. Prior analyses also indicate that sepsis is a common terminal pathway for patients with cancer.^29^ Severe debilitating strokes and dementia also accounted for a substantial number of sepsis-associated deaths, underscoring the increased risk of sepsis and poor outcomes conferred by frailty.^30,31^

Our findings are notable in light of many sepsis quality improvement initiatives that reported substantial decreases in mortality rates after implementation of sepsis care improvement initiatives.^32,33,34,35,36^ These studies imply that many sepsis-associated deaths are preventable. One possible explanation for this discrepancy is that improving sepsis care has already been a focus for the hospitals in our study. Another possible explanation is that analyses of performance improvement initiatives may overestimate their effect on mortality, because sepsis quality improvement initiatives place a strong emphasis on improving recognition of sepsis in addition to improving sepsis care. Improving recognition of sepsis leads to the inclusion of more subtle cases in the pool of patients with sepsis and gives an impression of declining overall mortality rates.^1,37,38,39^

### Limitations

Our study has important limitations. First, our analysis was limited to only 6 hospitals, several of which specialize in the care of patients with cancer and other complex conditions. Our findings thus may not be generalizable, particularly to low-resource settings within and outside the United States. Second, there are no universally accepted definitions for end-stage conditions and terminally ill patients.^40^ We chose to use Centers for Medicare & Medicaid Services hospice criteria because of their widespread use and clinical face validity, but they may not sufficiently capture patients with a high combined burden of comorbidities or those with advanced frailty marked by slowly progressive functional deterioration.^41^ Third, our study cohort is a sizable sample drawn from a finite population and we reported results using standard statistical procedures without finite sample correction. With finite sample correction, the standard errors for prevalence estimates are expected to be smaller. In addition, our study may be prone to type II errors from a lack of adequate power for all the comparisons we examined, such as the characteristics of patients who died with and without sepsis or patients with sepsis in academic hospitals vs community hospitals. Fourth, we did not assess whether better preventive care prior to hospitalization or more expeditious hospitalizations for infection or underlying conditions could have prevented death.^6,42^ In particular, one-quarter of patients who died with sepsis initially presented from long-term care facilities and it is unknown to what degree earlier recognition and care could have mitigated poor outcomes.^43^ This is an important topic for future research.

Determining the preventability of hospital deaths is invariably subjective and susceptible to multiple potential biases.^27^ For example, reviewers may be more likely to underassess preventability of deaths in their own hospitals because of a reluctance to appear critical of their colleagues. On the other hand, reviewers may be more likely to overassess preventability when outcomes are poor.^44^ Hindsight bias is a known issue that may predispose clinicians to label a poor outcome as preventable when retrospectively reviewing a case compared with caring for patients in the moment.^44^ There may also be important clinical nuances that cannot be ascertained retrospectively from medical records,^45^ particularly when trying to assess the promptness of recognizing and treating sepsis. Our preventability assessments could further be confounded by variations in the completeness and clarity of medical records between different hospitals in our study. We attempted to mitigate these limitations through intensive training sessions, structured medical record reviews, and reconciliation of training sets of reviews and achieved agreement levels similar to those in other studies of preventability,^20^ but agreement was still imperfect and remained below our goal threshold at 1 of the 3 study sites. However, we believe the residual subjectivity in our analyses underscores the complexity of patients dying with sepsis. Furthermore, prior work indicates that using a preventability measure with imperfect reliability may potentially overestimate the number of preventable cases.^46^ Our finding that only 1 in 8 sepsis-associated deaths is potentially preventable may therefore be conservative and challenges the overly simplistic assumption that most sepsis-associated deaths can be prevented with better hospital-based sepsis care.

## Conclusions

Sepsis was present in more than half of hospitalizations ending in death or terminal discharge to hospice in this cohort of patients from 6 US hospitals, and was the immediate cause of death in most of these cases. However, most underlying causes of death were associated with severe chronic comorbidities. One in 8 sepsis-associated deaths was potentially preventable through better hospital-based care, but only 1 in 25 sepsis-associated deaths was judged definitely or moderately preventable. Our findings do not diminish the importance of trying to prevent as many sepsis-associated deaths as possible, but rather underscore that most fatalities occur in medically complex patients with severe comorbid conditions. Further innovations in the prevention and care of underlying conditions may be necessary before a major reduction in sepsis-associated deaths can be achieved.

## Notes

Supplement. **eTable 1.** List of “End-Stage Comorbidities” Adapted From Hospice Eligibility Criteria from the Centers for Medicare & Medicaid Services **eTable 2.** Comparison of Hospice-Qualifying Terminal Conditions in Sepsis vs Non-Sepsis Patients Who Died In the Hospital or Were Discharged to Hospice **eTable 3.** Characteristics of Sepsis Patients Stratified by Academic vs Community Hospital **eTable 4.** Preventability of Death in Academic vs Community Hospitals **eTable 5.** Preventability of Death in Patients with Hospital-Onset Sepsis vs Sepsis Present on Admission **eTable 6.** Preventability of Death in Patients Where Sepsis Was the Immediate Cause of Death **eAppendix 1.** Data Collection Tool **eAppendix 2.** Criteria for Definite, Probable, and Possible Sepsis
