# Supplementary material for: Prevalence, Underlying Causes, and Preventability of Sepsis-Associated Mortality in US Acute Care Hospitals
Source: JAMA Netw Open. 2019 Feb 15;2(2):e187571. doi: 10.1001/jamanetworkopen.2018.7571 (PMC6484603; doi:10.1001/jamanetworkopen.2018.7571)
Supplement: Supplement. — eTable 1. List of “End-Stage Comorbidities” Adapted From Hospice Eligibility Criteria from the Centers for Medicare & Medicaid Services eTable 2. Comparison of Hospice-Qualifying Terminal Conditions in Sepsis vs Non-Sepsis Patients Who Died In the Hospital or Were Discharged to Hospice eTable 3. Characteristics of Sepsis Patients Stratified by Academic vs Community Hospital eTable 4. Preventability of Death in Academic vs Community Hospitals eTable 5. Preventability of Death in Patients with Hospital-Onset Sepsis vs Sepsis Present on Admission eTable 6. Preventability of Death in Patients Where Sepsis Was the Immediate Cause of Death eAppendix 1. Data Collection Tool eAppendix 2. Criteria for Definite, Probable, and Possible Sepsis [file jamanetwopen-2-e187571-s001.pdf]

Supplementary Online Content

Rhee C, Jones TM, Hamad Y, et al; Centers for Disease Control and Prevention (CDC) Prevention Epicenters Program. Prevalence, underlying causes, and preventability of sepsis-associated mortality in US acute care hospitals. *JAMA Netw Open.* 2019;2(2):e187571. doi:10.1001/jamanetworkopen.2018.7571

eTable 1. List of "End-Stage Comorbidities" Adapted From Hospice Eligibility Criteria from the Centers for Medicare & Medicaid Services
eTable 2. Comparison of Hospice-Qualifying Terminal Conditions in Sepsis vs Non-Sepsis Patients Who Died In the Hospital or Were Discharged to Hospice
eTable 3. Characteristics of Sepsis Patients Stratified by Academic vs Community Hospital
eTable 4. Preventability of Death in Academic vs Community Hospitals
eTable 5. Preventability of Death in Patients with Hospital-Onset Sepsis vs Sepsis Present on Admission
eTable 6. Preventability of Death in Patients Where Sepsis Was the Immediate Cause of Death
eAppendix 1. Data Collection Tool
eAppendix 2. Criteria for Definite, Probable, and Possible Sepsis

This supplementary material has been provided by the authors to give readers additional information about their work.

© 2019 Rhee C et al. *JAMA Network Open.*

eTable 1. List of “End-Stage Comorbidities” Adapted From Hospice Eligibility Criteria from the Centers for Medicare & Medicaid Services

Condition	Description
**Solid Cancer**	• Distant metastases, OR<br>• Progression from an earlier stage of disease to metastatic disease despite therapy (or patient declines further therapy)
**Hematologic Cancer**	• Progressive or refractory disease despite therapy, OR<br>• Patient refuses further therapy
**Dementia**	• Unable to perform activities of daily living without assistance and no meaningful verbal communication, AND<br>• ≥1 of the following within the past 12 months: aspiration pneumonia, urinary tract infection, sepsis, stage 3 or 4 decubitus ulcer, or albumin <2.5 g/dL
**Heart Failure**	• New York Heart Association Class IV heart failure (symptoms at rest) or ejection fraction ≤20%, AND<br>• Has been optimally treated for heart disease, or is not a candidate for a surgical procedure or has declined a procedure
**Human Immunodeficiency Virus**	• ≥1 serious Acquired Immunodeficiency Syndrome illness: central nervous system lymphoma, wasting, mycobacterium avium complex bacteremia, progressive multifocal leukoencephalopathy, systemic lymphoma, visceral Kaposi’s sarcoma unresponsive to therapy, toxoplasmosis unresponsive to therapy, or Cryptosporidium infection
**Liver Disease**	• International normalized ratio > 1.5 and albumin <2.5 g/dL AND<br>• At least one of the following: ascites, prior history of spontaneous bacterial peritonitis, hepatorenal syndrome, hepatic encephalopathy, recurrent variceal bleeding, or awaiting liver transplant
**Chronic Lung Disease**	• Dyspnea at rest and ≥2 emergency department visits or hospitalizations within the prior 12 months for respiratory infection or pulmonary infections, AND<br>• Hypoxemia at rest on room air (oxygen saturation ≤88% or partial pressure of oxygen ≤55 mmHg) or hypercapnia with partial pressure of carbon dioxide ≥50 mmHg
**Chronic Renal Disease**	• Creatinine clearance of <10 cc/min, or serum creatinine >8.0 mg/dL AND<br>• Patient not seeking dialysis or renal transplant
**Prior Stroke**	• Unable to care for self (requiring hospital or skilled nursing care) AND<br>• Weight loss >10% in past 6 months or serum albumin <2.5 g/dL or current history of pulmonary aspiration or severe dysphagia preventing adequate oral nutrition and declining artificial nutrition and hydration
**Coma**	• Documented comatose state lasting for 3 or more days
**Amyotrophic Lateral Scelorosis**	• Impaired respiratory status with vital capacity <30% of normal or dyspnea at rest, OR<br>• Wheelchair or bed-bound status and/or need for major assistance by caretaker for activities of daily living, OR<br>• ≥2 in the past 12 months: aspiration pneumonia, urinary tract infection, sepsis, stage 3 or 4 decubitus ulcer

© 2019 Rhee C et al. *JAMA Network Open.*

eTable 2. Comparison of Hospice-Qualifying Terminal Conditions in Sepsis vs Non-Sepsis Patients Who Died In the Hospital or Were Discharged to Hospice

Hospice-Qualifying Condition	Sepsis Present (n=300)	No Sepsis (n=268)	p-value
**Solid Cancer**	60 (20.0%)	90 (33.6%)	<0.001
**Hematologic Cancer**	16 (5.3%)	12 (4.5%)	0.64
**Dementia**	15 (5.0%)	6 (2.2%)	0.08
**Heart Failure**	10 (3.3%)	13 (4.9%)	0.36
**HIV**	1 (0.3%)	0 (0%)	0.35
**Liver Disease**	4 (1.3%)	6 (2.2%)	0.41
**Chronic Lung Disease**	12 (4.0%)	11 (4.1%)	0.95
**Chronic Renal Disease**	1 (0.3%)	3 (1.1%)	0.26
**Prior Stroke**	12 (4.0%)	0 (0%)	<0.001
**Coma**	0 (0%)	0 (0%)	-
**ALS**	0 (0%)	0 (0%)	-
Any Terminal Comorbidity	*121 (40.3%)*	*135 (50.4%)*	*0.02*

© 2019 Rhee C et al. *JAMA Network Open.*

eTable 3. Characteristics of Sepsis Patients Stratified by Academic vs Community Hospital

Characteristic	Community Hospital (n=154)	Academic Hospital (n=146)	p-value
Mean Age (SD), years	76.8 (14.5)	64.4 (14.8)	<0.001
Male Sex (N,%)	83 (53.9%)	62 (42.5%)	0.05
Race / Ethnicity			0.05
White (N,%)	126 (81.8%)	100 (68.5%)	
Black (N,%)	19 (12.3%)	35 (24.0%)	
Hispanic (N,%)	5 (3.3%)	5 (3.4%)	
Other (N,%)	4 (2.6%)	6 (4.1%)	
Preadmission Location			<0.001
Home (N,%)	95 (61.7%)	127 (87.0%)	
Facility (N,%)	59 (38.3%)	19 (13.0%)	
Admitting Service			<0.001
Medical (N,%)	147 (95.5%)	110 (75.3%)	
Surgical (N,%)	4 (2.6%)	27 (18.5%)	
Other (N,%)	3 (2.0%)	9 (6.2%)	
Type of Admission			0.79
Emergent (N,%)	149 (96.8%)	139 (95.2%)	
Elective Surgery (N,%)	3 (2.0%)	4 (2.7%)	
Elective Medical (N,%)	2 (1.3%)	3 (2.1%)	
DNR/DNI on Admission (N,%)	48 (31.2%)	19 (13.0%)	<0.001
Required ICU Admission (N,%)	106 (68.8%)	103 (71.0%)	0.68
Unit Location at Death			<0.001
ICU (N,%)	65 (53.3%)	86 (71.1%)	
Non-ICU Ward (N,%)	57 (46.7%)	34(28.1%)	
Emergency Department (N,%)	0 (0%)	1 (0.8%)	
Comorbidities			
Solid Cancer (N,%)	33 (21.4%)	53 (36.3%)	0.005
Hematologic Cancer (N,%)	7 (4.6%)	24 (16.4%)	<0.001
Dementia (N,%)	42 (27.3%)	4 (2.7%)	<0.001
Heart Failure (N,%)	34 (23.3%)	39 (25.3%)	0.68
Liver Disease (N,%)	6 (3.9%)	13 (8.9%)	0.08
Chronic Lung Disease (N,%)	37 (24.0%)	34 (23.3%)	0.88
Chronic Renal Disease (N,%)	45 (29.2%)	30 (20.6%)	0.08
Prior Stroke (N,%)	29 (18.8%)	16 (11.0%)	0.06
Coronary Disease (N,%)	55 (35.7%)	37 (25.3%)	0.05
Diabetes (N,%)	48 (31.2%)	54 (37.0%)	0.29
Substance Abuse (N,%)	6 (3.9%)	8 (5.5%)	0.52
Hypertension (N,%)	101 (65.6%)	90 (61.6%)	0.48
Atrial Fibrillation (N,%)	48 (31.2%)	26 (17.8%)	0.007
Hospitalization within:			
Prior Year (N,%)	105 (68.2%)	80 (54.8%)	0.02
Prior 60 days (N,%)	68 (44.2%)	57 (39.0%)	0.37
Median Hospital LOS (IQR), days	7 (4-13)	11 (6-20)	0.001
Median ICU LOS (IQR), days	4 (2-8)	5 (3-14)	0.03
Death (N,%)	122 (79.2%)	121 (82.9%)	0.42
Hospice (N,%)	32 (20.8%)	25 (17.1%)	

Abbreviations: ICU = intensive care unit, DNR =do not resuscitate, DNI = do not intubate, LOS = length of stay

© 2019 Rhee C et al. *JAMA Network Open.*

eTable 4. Preventability of Death in Academic vs Community Hospitals

Preventability Categories	Community Hospital (n=154)	Academic Hospital (n=146)
**1 (Definitely Preventable)**	3 (2.0%)	1 (0.7%)
**2 (Moderately Likely to be Preventable)**	4 (2.6%)	3 (2.1%)
**3 (Possibly Preventable)**	14 (9.1%)	11 (7.5%)
**4 (Unlikely to be Preventable)**	18 (11.7%)	27 (18.5%)
**5 (Moderately Likely to Not be Preventable)**	47 (30.5%)	43 (29.5%)
**6 (Definitely Not Preventable)**	68 (44.2%)	61 (41.8%)

*Note: the overall p-value for comparison of preventability ratings between community and academic hospitals was non-significant (p=0.60).

© 2019 Rhee C et al. *JAMA Network Open.*

eTable 5. Preventability of Death in Patients with Hospital-Onset Sepsis vs Sepsis Present on Admission

Preventability Categories	Sepsis Present-on-Admission (n=221)	Hospital-Onset Sepsis (n=79)
**1 (Definitely Preventable)**	2 (0.9%)	2 (2.5%)
**2 (Moderately Likely to be Preventable)**	5 (2.3%)	2 (2.5%)
**3 (Possibly Preventable)**	21 (9.5%)	4 (5.1%)
**4 (Unlikely to be Preventable)**	27 (12.2%)	18 (22.8%)
**5 (Moderately Likely to Not be Preventable)**	67 (30.3%)	23 (29.1%)
**6 (Definitely Not Preventable)**	99 (44.8%)	30 (38.0%)

*Note: the overall p-value for comparison of preventability ratings between hospital-onset sepsis and sepsis present-on-admission was non-significant (p=0.19).

© 2019 Rhee C et al. *JAMA Network Open.*

eTable 6. Preventability of Death in Patients Where Sepsis Was the Immediate Cause of Death

Preventability Categories	Sepsis as Immediate Cause of Death (n=198)
**1 (Definitely Preventable)**	4 (2.0%)
**2 (Moderately Likely to be Preventable)**	6 (3.0%)
**3 (Possibly Preventable)**	20 (10.1%)
**4 (Unlikely to be Preventable)**	33 (16.7%)
**5 (Moderately Likely to Not be Preventable)**	60 (30.3%)
**6 (Definitely Not Preventable)**	75 (37.9%)

© 2019 Rhee C et al. *JAMA Network Open.*

SECTION I. BASIC ID AND BACKGROUND INFORMATION

Study ID/MRN ____________________

Hospital

- ◯ BWH (Main Academic Hospital)
- ◯ BWH Faulkner (Community
- ◯ Affiliate) Duke (Main Academic
- ◯ Hospital) Duke (Community
- ◯ Affiliate)
- ◯ Wash U (Main Academic
  Hospital) Wash U (Community
  Affiliate)

Admit Date ____________________

Date of Death or Discharge to Hospice ____________________

Discharge Disposition

- ◯ Death
- ◯ Hospice (Home)
- ◯ Hospice
- ◯ (Facility)
  Not death or hospice (incorrectly flagged chart)

If Death - Location at Time of Death

- ◯ ED (or ED
- ◯ Observation) Inpatient
- ◯ Ward
- ◯ ICU
  Inpatient hospice unit

If Hospice - How Long After Discharge Did the Patient Die?

- ◯ < =1 week
- ◯ >1 week
- ◯ Unknown
- ◯ Patient still alive

Preadmission Location

If patient was already on hospice prior to admission, please continue ONLY if patient died during this hospitalization. If patient was admitted and discharged to hospice, please stop here.

- ◯ Home (includes assisted
- ◯ living) Long-term care facility
- ◯ Acute rehabilitation facility
- ◯ Hospice --> STOP ABSTRACTION IF PATIENT WAS DISCHARGED BACK TO HOSPICE. CONTINUE ONLY IF PATIENT DIED DURING HOSPITALIZATION.
- ◯ Other

Patient Age on Admission (in Years) ____________________

Sex

- ◯ Male
- ◯ Female

Race

- ◯ White
- ◯ Black
- ◯ Asian
- ◯ American Indian / Alaska Native
- ◯ Native Hawaiian or Other Pacific
- ◯ Mixed Race
- ◯ Other
- ◯ Unknown

© 2019 Rhee C et al. *JAMA Network Open.*

Ethnicity

- ◯ Hispanic or Latino
- ◯ Not Hispanic or Latino
- ◯ Unknown

Was the Patient Transferred from an Outside Hospital?

- ◯ No
- ◯ Yes, Outside Hospital Emergency Department
- ◯ Yes, Outside Hospital Ward or ICU

Number of Inpatient Hospitalizations in the Past Year (365 days) - not including hospitalization of interest. (Please attempt to include hospitalizations outside and within your healthcare system as best as can be determined from H+P, discharge summaries, consult notes, etc.)

____

Date of Last Hospital Discharge (leave blank if unknown)

____

Primary Admitting Service

- ◯ Medical (including MICU, Heme/Onc,
- ◯ Cardiology) Surgical (including SICU and surgical subspecialties)
- ◯ Neurology (including neuro-ICU. Primary neurosurgery patients should be listed under Surgical)
- ◯ Obstetrics / Gynecology (*For Gynecology/Oncology patients, please list under Surgical)
- ◯ Emergency Medicine (i.e., if patient died in ED)
- ◯ Other

Reason for / Type of Admission

- ◯ Acute illness
- ◯ Elective surgery
- ◯ Elective medical admit
- ◯ Other

Did the Patient Require ICU Admission?

- ◯ Y
- ◯ N

Date of ICU Admission (If multiple admits, list only first date)

____

ICU Length of Stay (If multiple ICU stays, sum all days)

____

SECTION II. COMORBIDITIES

Was the patient immunosuppressed? (Check all that apply)

- ☐ Not immunosuppressed
- ☐ Active hematologic malignancy (e.g. AML, CLL)
- ☐ Active solid malignancy requiring chemotherapy within prior 60 days
- ☐ Stem cell transplant requiring immune-modulating medications
- ☐ Solid organ transplant requiring immune-modulating medications
- ☐ HIV infection
- ☐ Chronic inflammatory condition and receiving immune-modulating medications within prior 60 days (if steroids alone, requires equivalent of >=20 mg/day of prednisone for at least one month)

© 2019 Rhee C et al. *JAMA Network Open.*

Were any of the following potentially "end-stage conditions" present on admission?
(Choose all that apply)

No potentially end-stage conditions

- ☐ Solid Cancer
- ☐ Hematologic Cancer (acute or chronic leukemia or lymphoma)
- ☐ Dementia
- ☐ Heart Failure
- ☐ HIV Disease
- ☐ Liver
- ☐ Disease
- ☐ Chronic Pulmonary
- ☐ Disease Chronic Renal
- ☐ Disease Prior Stroke
- ☐ Coma
- ☐ Amyotrophic Lateral Sclerosis

Solid Cancer

- ○ Distant metastases, OR progression from an earlier stage of disease to metastatic disease despite therapy (or patient declines further therapy)
- ○ Solid Cancer present but above criteria not met

Hematologic Cancer

- ○ Progressive or refractory disease despite therapy, OR patient refuses further therapy
- ○ Hematologic cancer present, but above criteria not met

Dementia

- ○ Unable to perform ADLs without assistance and no meaningful verbal communication, AND >=1 of the following within the past 12 months: aspiration pneumonia, UTI, sepsis, stage 3 or 4 decubitus ulcer, albumin < 2.5 g/dL
- ○ Dementia present but above criteria not met

Heart Failure

- ○ [New York Heart Association Class IV heart failure (symptoms at rest) or ejection fraction < =20%] AND [Has been optimally treated for heart disease, or is not a candidate for a surgical procedure or has declined a procedure]
- ○ Heart failure present, but above criteria not met

HIV Disease

- ○ >=1 serious AIDS illness: CNS lymphoma, wasting, mycobacterium avium complex bacteremia, progressive multifocal leukoencephalopathy, systemic lymphoma, visceral kaposi's sarcoma unresponsive to therapy, toxoplasmosis unresponsive to therapy, cryptosporidium infection
- ○ HIV present, but above criteria not met

Liver Disease

- ○ INR >1.5 and albumin < 2.5 g/dL AND at least one of: (Ascites) (Prior history of spontaneous bacterial peritonitis) (Hepatorenal syndrome) (Hepatic encephalopathy) (Recurrent variceal bleeding) (Awaiting liver transplant)
- ○ Liver disease present, but above criteria not met

Chronic Pulmonary Disease

- ○ [Dyspnea at rest and >=2 ED visits or hospitalizations within prior 12 months for respiratory failure or pulmonary infections] AND [(Hypoxemia at rest on room air - O2 sat < =88% or PaO2 < =55 mmHg, or Hypercapnia with PCO2 >=50 mmHg]
- ○ Chronic pulmonary disease present, but above criteria not met

© 2019 Rhee C et al. *JAMA Network Open.*

Chronic Renal Disease

- ◯ Creatinine clearance of < 10 cc/min, or Serum creatinine >8.0 mg/dL AND patient not seeking dialysis or renaltransplant
- ◯ Chronic renal disease present but above criteria not met

Prior Stroke

- ◯ (Unable to care for self (requiring hospital or skilled nursing care) AND weight loss >10% in past 6 months) OR (serum albumin < 2.5 g/dL) OR (Current history of pulmonary aspiration or severe dysphagia preventing adequate oral nutrition and declining artificial nutrition and hydration)
- ◯ History of prior stroke, but above criteria not met

Coma

- ◯ Documented comatose state lasting for 3 or more days
- ◯ Comatose but above criteria not met (i.e. < 3 days)

Amyotrophic Lateral Sclerosis

- ◯ (Impaired respiratory status with vital capicity < 30% of normal or dyspnea at rest) OR (wheelchair or bed-bound status and/or need for major assistance by caretaker for ADLs) OR (>=2 in the past 12 months: aspiration pneumonia, UTI, sepsis, stage 3 or 4 decubutis ulcer)
- ◯ ALS but above criteria not present

Other Comorbidities (check all that apply)

- ☐ Alcohol Abuse
- ☐ Atrial fibrillation
- ☐ Coronary heart
- ☐ disease Diabetes
- ☐ Drug Abuse
- ☐ Hypertension
- ☐ Peripheral vascular
- ☐ disease Rheumatologic disease

SECTION III. CAUSE OF DEATH

Was the IMMEDIATE cause of death an infection?
(Or, if patient transitioned to CMO / hospice, was infection the immediate condition that led to the patient's deterioration?)

Note: the IMMEDIATE cause of death is the final disease, injury, or complication directly causing death. It does NOT mean the mechanism of death or terminal event (for example, cardiac arrest or respiratory arrest - as these are not specific and merely attest to the fact of death).

- ◯ Infection
- ◯ Not infection

© 2019 Rhee C et al. *JAMA Network Open.*

What was the Likely Source of Infection? (Choose one).

If there were multiple sources of infection, please choose the one that was likely to be the most severe infection.

- ◯ Primary Bacteremia
- ◯ Pneumonia / Pulmonary
- ◯ Urinary / Genitourinary
- ◯ GI / Intra-abdominal / Hepatic
- ◯ Skin / Soft Tissue
- ◯ Central Nervous System
- ◯ Gynecologic
- ◯ Catheter
- ◯ Endovascular / Cardiovascular
- ◯ Febrile Neutropenia with no source identified
- ◯ Unknown Source
- ◯ Other

Other Infection Source ________________________

What was the causative pathogen type?

- ◯ Bacterial - gram-positive
- ◯ Bacterial -gram-negative
- ◯ Bacterial -other
- ◯ Fungal - yeast
- ◯ Fungal - mold
- ◯ Fungal - other
- ◯ Viral - influenza
- ◯ Viral - non-influenza respiratory virus
- ◯ Viral - herpesvirus (HSV, VZV, CMV)
- ◯ Viral - other
- ◯ Protozoal/Parasitic
- ◯ Mycobacterial
- ◯ Unknown
- ◯ Other
- ◯

Other Pathogen Type ________________________

Not infection: What was the IMMEDIATE cause of death (or condition triggering hospice)?

- ◯ Cardiac
- ◯ Pulmonary
- ◯ GI / Liver
- ◯ Neurologic
- ◯ Trauma
- ◯ Hemorrhage
- ◯ Renal
- ◯ Failure
- ◯ Unknown
- ◯ Progressive cancer, with no other specific cause
- ◯ Patient discharged to hospice but was already on hospice prior to admission; current hospitalization did not "trigger" new deterioration
- ◯ Other

Other Cause of Death ________________________

Cardiovascular Death:

- ◯ Myocardial infarction
- ◯ Arrhythmia
- ◯ Heart failure
- ◯ Tamponade
- ◯ Other cardiovascular cause

© 2019 Rhee C et al. *JAMA Network Open.*

Pulmonary Death

- ◯ ARDS
- ◯ Airway compression
- ◯ Aspiration
- ◯ Asthma exacerbation
- ◯ COPD exacerbation
- ◯ InterstitialLungDisease
- ◯ Pneumothorax
- ◯ Pulmonary embolism
- ◯ Pulmonary edema
- ◯ Other Pulmonary

GI / Liver Death

- ◯ Acute liver failure
- ◯ GI bleed
- ◯ Pancreatits
- ◯ Other GI or Liver cause

Neurologic Death

- ◯ Hypoxic brain injury
- ◯ Ischemic stroke
- ◯ Intracerebral or subarachnoid hemorrhage
- ◯ Other neurologic death

What was the UNDERLYING cause of death (or cause of decompensation leading to hospice / CMO)

Note: The Underlying cause of death is the disease or injury that initiated the chain of events that led directly or inevitably to death. For example, sepsis is typically not an underlying cause of death. But for a patient with AML getting chemotherapy who develops Klebsiella bacteremia and mellitus septic shock, AML would be the UNDERLYING cause of death.

- ◯ Solid Cancer
- ◯ Heme Cancer
- ◯ Chronic Heart Disease
- ◯ Chronic Liver Disease
- ◯ Chronic Pulm Disease
- ◯ Chronic Renal Disease
- ◯ Chronic Alcoholism or Substance Abuse
- ◯ Dementia
- ◯ Diabetes
- ◯ HIV infection
- ◯ Peripheral vascular disease
- ◯ Psychiatric
- ◯ disease Stroke
- ◯ Trauma or Injury
- ◯ Hypertension
- ◯ Unknown
- ◯ Other

Other Underlying Cause of Death ____________________________

SECTION IV - Part A. INFECTION / SEPSIS AS CAUSE OF DEATH - Please only complete this section if infection was the immediate cause of death.

Approximate date of infection onset (m-d-y); if infection started prior to hospitalization, list date of admission as date of onset ____________________________

Cardiovascular SOFA Score during most acutely ill 24-hour period while infected

"Most acutely ill period" can generally be easily identified from scanning progress notes / flowsheets (i.e., time period when patient was on max pressors or vent settings). This is often, but not always, the day of death. Most patients with infection/sepsis as a cause of death will far exceed SOFA score rise by >=2.

- ◯ 0 points: MAP >= 70mmHg
- ◯ 1 point: MAP < 70 mmHg
- ◯ 2 points: Dopamine < =5 mcg/kg/min or Dobutamine (any dose), or any dose Phenylephrine or Vasopressin
- ◯ 3 points: Dopamine > 5 mcg/kg/min or Epinephrine < = 0.1 mcg/kg/min or Norepinephrine < = 0.1 mcg/kg/min
- ◯ 4 points: Dopamine > 15 mcg/kg/min or Epinephrine >0.1 mcg/kg/min or Norepinephrine >0.1 mcg/kg/min
- ◯ Missing

© 2019 Rhee C et al. *JAMA Network Open.*

Neuro SOFA Score during most acutely ill 24-hour period while infected

- ◯ 0 points: GCS 15
- ◯ 1 point: GCS 13-14, mildly confused, or light sedation while intubated (i.e. RASS -2 or +2)
- ◯ 2 points: GCS 10-12, moderately confused, or moderately sedated while intubated (i.e. RASS -3 or +3 or +4)
- ◯ 3 points: GCS 6-9, severely obtunded, or deeply sedated while intubated (i.e., RASS -4 or -5)
- ◯ 4 points: GCS < 6, virtually comatose / severe neurologic injury
- ◯ Missing

Respiratory SOFA Score during most acutely ill 24-hour period while infected (use PaO2/FiO2 ratio if available; otherwise, use SaO2/FiO2 ratio).

To estimate FiO2 for patients who are non-intubated or not on BIPAP/CPAP, a rough conversion is as follows: RA = 0.21, 1 L NC = 0.24, 2 L NC = 0.28, 3 L NC = 0.32, 4 L NC = 0.36, 5 L NC = 0.40, 6 L NC = 0.44). A patient on 100% non-rebreather can be estimated as having an FiO2 of 1.0. Patients on venti or face masks usually have FiO2 listed (i.e, 60% Face Mask), as do patients on High-Flow Nasal Canula, so please use listed FiO2 in that scenario.

- ◯ 0 points: PaO2/FiO2 ratio >=400, or SaO2/FiO2 ratio >301
- ◯ 1 point: PaO2/FiO2 ratio 300-399, or SaO2/FiO2 ratio 221-301
- ◯ 2 points: PaO2/FiO2 ratio < 300, or SaO2/FiO2 ratio < 221
- ◯ 3 points: PaO2/FiO2 ratio 100-199 (or SaO2/FiO2 ratio 67-141) AND mechanically ventilated
- ◯ 4 points: PaO2/FiO2 ratio < 100 (or SaO2/FiO2 ratio < 67) AND mechanically ventilated
- ◯ Missing

Renal SOFA Score during most acutely ill 24-hour period while infected

- ◯ 0 points: Cr < 1.2
- ◯ 1 point: Cr 1.2-1.9
- ◯ 2 points: Cr 2.0-3.4
- ◯ 3 points: Cr 3.5-4.9, or urine output < 500 cc/day
- ◯ 4 points: Cr >5.0 or urine output < 200 cc/day
- ◯ Missing

Liver SOFA Score during most acutely ill 24-hour period while infected

- ◯ 0 points: Bilirubin < 1.2
- ◯ 1 point: Bilirubin 1.2-1.9
- ◯ 2 points: Bilirubin 2.0-5.9
- ◯ 3 points: Bilirubin 6.0-11.9
- ◯ 4 points: Bilirubin>=12.0
- ◯ Missing

Platelet SOFA Score during most acutely ill 24-hour period while infected

- ◯ 0 points: Platelets >=150
- ◯ 1 point: Platelets 100-149
- ◯ 2 points: Platelets 50-99
- ◯ 3 points: Platelets 20-49
- ◯ 4 points: Platelets < 20
- ◯ Missing

Total SOFA score during most acutely ill 24-hour period

____

Baseline Cardiovascular SOFA Score (all baseline values during a 24-hour period before infection onset)

Baseline organ dysfunction can usually be estimated by looking at prior hospitalizations (i.e., to estimate baseline creatinine) for patients with community-acquired sepsis, orduring earlier timeperiods during hospitalization for patients with hospital-acquired sepsis. If baseline organ dysfunction is unknown, assume a score of 0 for that category.

- ◯ 0 points: MAP >= 70mmHg
- ◯ 1 point: MAP < 70 mmHg
- ◯ 2 points: Dopamine < =5 mcg/kg/min or Dobutamine (any dose), or any dose Phenylephrine or Vasopressin
- ◯ 3 points: Dopamine > 5 mcg/kg/min or Epinephrine < = 0.1 mcg/kg/min or Norepinephrine < = 0.1 mcg/kg/min
- ◯ 4 points: Dopamine > 15 mcg/kg/min or Epinephrine >0.1 mcg/kg/min or Norepinephrine >0.1 mcg/kg/min
- ◯ Missing

© 2019 Rhee C et al. *JAMA Network Open.*

Baseline Neuro SOFA Score (all baseline values during a 24-hour period before infection onset)

- ◯ 0 points: GCS 15
- ◯ 1 point: GCS 13-14, mildly confused, or light sedation while intubated (i.e. RASS -2 or +2)
- ◯ 2 points: GCS 10-12, moderately confused, or moderately sedated while intubated (i.e. RASS -3 or +3 or +4)
- ◯ 3 points: GCS 6-9, severely obtunded, or deeply sedated while intubated (i.e., RASS -4 or -5)
- ◯ 4 points: GCS < 6, virtually comatose / severe neurologic injury
- ◯ Missing

Baseline Respiratory SOFA Score (use PaO2/FiO2 ratio if available; otherwise, use SaO2/FiO2 ratio) (all baseline values during a 24-hour period before SaO2/FiO2 infection onset)

To estimate FiO2 for patients who are non-intubated or not on BIPAP/CPAP, a rough conversion is as SaO2/FiO2 follows: RA = 0.21, 1 L NC = 0.24, 2 L NC = 0.28, 3 L NC = 0.32, 4 L NC = 0.36, 5 L NC = 0.40, 6 L NC = 0.44). A patient on 100% non-rebreather can be estimated as having an FiO2 of 1.0. Patients on venti or face masks usually have FiO2 listed (i.e, 60% Face Mask), as do patients on High-Flow Nasal Canula, so please use listed FiO2 in that scenario.

- ◯ 0 points: PaO2/FiO2 ratio >=400, or SaO2/FiO2 ratio >301
- ◯ 1 point: PaO2/FiO2 ratio 300-399, or ratio 221-301
- ◯ 2 points: PaO2/FiO2 ratio < 300, or SaO2/FiO2 ratio < 221
- ◯ 3 points: PaO2/FiO2 ratio 100-199 (or ratio 67-141) AND mechanically ventilated
- ◯ 4 points: PaO2/FiO2 ratio < 100 (or SaO2/FiO2 ratio < 67) AND mechanically ventilated
- ◯ Missing

Baseline Renal SOFA Score (all baseline values during a 24-hour period before infection onset)

- ◯ 0 points: Cr < 1.2
- ◯ 1 point: Cr 1.2-1.9
- ◯ 2 points: Cr 2.0-3.4
- ◯ 3 points: Cr 3.5-4.9, or urine output < 500 cc/day
- ◯ 4 points: Cr >5.0 or urine output < 200 cc/day
- ◯ Missing

Baseline Liver SOFA Score (all baseline values during a 24-hour period before infection onset)

- ◯ 0 points: Bilirubin < 1.2
- ◯ 1 point: Bilirubin 1.2-1.9
- ◯ 2 points: Bilirubin 2.0-5.9
- ◯ 3 points: Bilirubin 6.0-11.9
- ◯ 4 points: Bilirubin>=12.0
- ◯ Missing

Baseline Platelet SOFA Score (all baseline values during a 24-hour period before infection onset)

- ◯ 0 points: Platelets >=150
- ◯ 1 point: Platelets 100-149
- ◯ 2 points: Platelets 50-99
- ◯ 3 points: Platelets 20-49
- ◯ 4 points: Platelets < 20
- ◯ Missing

Baseline SOFA score (all baseline values during a 24-hour period before infection onset) ____________________________

© 2019 Rhee C et al. *JAMA Network Open.*

Did the terminal infection qualify as sepsis, as defined by an acute rise in SOFA score by >=2 attributable to infection?

For patients transferred from an outside hospital, If infection does not meet sepsis/SOFA criteria at the current hospitalization, but patient clearly met sepsis criteria PRIOR to transfer (i.e, based on OSH notes documenting septic shock requiring pressors), please take that into account for this question.

- ◯ Definite sepsis (i.e., clear source of infection and no other obvious contributors to organ dysfunction)
- ◯ Probable sepsis (i.e.,clear or probable infection; other factors may have contributed to organ dysfunction but infection was likely the primary driver)
- ◯ Possible sepsis (i.e., no definitive source of infection identified but patient treated for infection/sepsis and no other obvious cause of organ dysfunction, OR infection likely to be present and may have been one of several possible contributors to organ dysfunction)
- ◯ Not sepsis

SECTION IV - Part B. INFECTION / SEPSIS ELSEWHERE DURING HOSPITALIZATION. Please only complete this section if infection was NOT the immediate cause of death.

If infection was not the primary cause of death (or reason for hospice), did the patient have an acute infection during hospitalization? (For patients whose course started at an outside hospital, please include infections diagnosed and first treated at the outside hospital prior to transfer).

- ◯ Definite infection
- ◯ Probable infection
- ◯ Possible infection
- ◯ No infection

What was the Likely Source of Infection? (Choose all that apply)

- ☐ Primary Bacteremia
- ☐ Pneumonia/Pulmonary
- ☐ Urinary/Genitourinary
- ☐ GI/Intra-abdominal / Hepatic
- ☐ Skin/Soft Tissue
- ☐ CNS
- ☐ Gynecologic
- ☐ Catheter
- ☐ Endovascular / Cardiovascular
- ☐ Febrile Neutropenia with no source identified
- ☐ Unknown Source
- ☐ Other

If other source of infection, please list ______________________________

Approximate date of infection onset (m-d-y); if infection started prior to hospitalization, list date of admission as date of onset ______________________________

Cardiovascular SOFA Score during most acutely ill 24-hour period while infected

"Most acutely ill period" can generally be easily identified from scanning progress notes / flowsheets (i.e., time period when patient was on max pressors or vent settings). This is often, but not always, the day of death. Most patients with infection/sepsis as a cause of death will far exceed SOFA score rise by >=2.

- ◯ 0 points: MAP >= 70mmHg
- ◯ 1 point: MAP < 70 mmHg
- ◯ 2 points: Dopamine < =5 mcg/kg/min or Dobutamine (any dose), or any dose Phenylephrine or Vasopressin
- ◯ 3 points: Dopamine > 5 mcg/kg/min or Epinephrine < = 0.1 mcg/kg/min or Norepinephrine < = 0.1 mcg/kg/min
- ◯ 4 points: Dopamine > 15 mcg/kg/min or Epinephrine >0.1 mcg/kg/min or Norepinephrine >0.1 mcg/kg/min
- ◯ Missing

© 2019 Rhee C et al. *JAMA Network Open.*

Neuro SOFA Score during most acutely ill 24-hour period while infected

- ◯ 0 points: GCS 15
- ◯ 1 point: GCS 13-14, mildly confused, or light sedation while intubated (i.e. RASS -2 or +2)
- ◯ 2 points: GCS 10-12, moderately confused, or moderately sedated while intubated (i.e. RASS -3 or +3 or +4)
- ◯ 3 points: GCS 6-9, severely obtunded, or deeply sedated while intubated (i.e., RASS -4 or -5)
- ◯ 4 points: GCS < 6, virtually comatose / severe neurologic injury
- ◯ Missing

Respiratory SOFA Score during most acutely ill 24-hour period while infected (use PaO2/FiO2 ratio if available; otherwise, use SaO2/FiO2 ratio)

To estimate FiO2 for patients who are non-intubated or not on BIPAP/CPAP, a rough conversion is as follows: RA = 0.21, 1 L NC = 0.24, 2 L NC = 0.28, 3 L NC = 0.32, 4 L NC = 0.36, 5 L NC = 0.40, 6 L NC = 0.44). A patient on 100% non-rebreather can be estimated as having an FiO2 of1.0. Patients on venti or face masks usually have FiO2 listed (i.e, 60% Face Mask), as do patients on High-Flow Nasal Canula, so please use listed FiO2 in that scenario.

- ◯ 0 points: PaO2/FiO2 ratio >=400, or SaO2/FiO2 ratio >301
- ◯ 1 point: PaO2/FiO2 ratio 300-399, or SaO2/FiO2 ratio 221-301
- ◯ 2 points: PaO2/FiO2 ratio < 300, or SaO2/FiO2 ratio < 221
- ◯ 3 points: PaO2/FiO2 ratio 100-199 (or SaO2/FiO2 ratio 67-141) AND mechanically ventilated
- ◯ 4 points: PaO2/FiO2 ratio < 100-199 (or SaO2/FiO2 ratio < 67) AND mechanically ventilated
- ◯ Missing

Renal SOFA Score during most acutely ill 24-hour period while infected

- ◯ 0 points: Cr < 1.2
- ◯ 1 point: Cr 1.2-1.9
- ◯ 2 points: Cr 2.0-3.4
- ◯ 3 points: Cr 3.5-4.9, or urine output < 500 cc/day
- ◯ 4 points: Cr >5.0 or urine output < 200 cc/day
- ◯ Missing

Liver SOFA Score during most acutely ill 24-hour period while infected

- ◯ 0 points: Bilirubin < 1.2
- ◯ 1 point: Bilirubin 1.2-1.9
- ◯ 2 points: Bilirubin 2.0-5.9
- ◯ 3 points: Bilirubin 6.0-11.9
- ◯ 4 points: Bilirubin>=12.0
- ◯ Missing

Platelet SOFA Score during most acutely ill 24-hour period while infected

- ◯ 0 points: Platelets >=150
- ◯ 1 point: Platelets 100-149
- ◯ 2 points: Platelets 50-99
- ◯ 3 points: Platelets 20-49
- ◯ 4 points: Platelets < 20
- ◯ Missing

Total SOFA score during most acutely ill 24-hour period

____

Baseline Cardiovascular SOFA Score (all baseline values during a 24-hour period before infection onset)

Baseline organ dysfunction can usually be estimated by looking at prior hospitalizations (i.e., to estimate baseline creatinine) for patients with community-acquired sepsis, orduring earlier timeperiods during hospitalization for patients with hospital-acquired sepsis. If baseline organ dysfunction is unknown, assume a score of 0 for that category.

- ◯ 0 points: MAP >= 70mmHg
- ◯ 1 point: MAP < 70 mmHg
- ◯ 2 points: Dopamine < =5 mcg/kg/min or Dobutamine (any dose), or any dose Phenylephrine or Vasopressin
- ◯ 3 points: Dopamine > 5 mcg/kg/min or Epinephrine < = 0.1 mcg/kg/min or Norepinephrine < = 0.1 mcg/kg/min
- ◯ 4 points: Dopamine > 15 mcg/kg/min or Epinephrine >0.1 mcg/kg/min or Norepinephrine >0.1 mcg/kg/min
- ◯ Missing

© 2019 Rhee C et al. *JAMA Network Open.*

Baseline Neuro SOFA Score (all baseline values during a 24-hour period before infection onset)

- ◯ 0 points: GCS 15
- ◯ 1 point: GCS 13-14, mildly confused, or light sedation while intubated (i.e. RASS -2 or +2)
- ◯ 2 points: GCS 10-12, moderately confused, or moderately sedated while intubated (i.e. RASS -3 or +3 or +4)
- ◯ 3 points: GCS 6-9, severely obtunded, or deeply sedated while intubated (i.e., RASS -4 or -5)
- ◯ 4 points: GCS < 6, virtually comatose / severe neurologic injury
- ◯ Missing

Baseline Respiratory SOFA Score (use PaO2/FiO2 ratio if available; otherwise, use SaO2/FiO2 ratio) (all baseline values during a 24-hour period before SaO2/FiO2 infection onset)

To estimate FiO2 for patients who are non-intubated or not on BIPAP/CPAP, a rough conversion is as SaO2/FiO2 follows: RA = 0.21, 1 L NC = 0.24, 2 L NC = 0.28, 3 L NC = 0.32, 4 L NC = 0.36, 5 L NC = 0.40, 6 L NC = 0.44). A patient on 100% non-rebreather can be estimated as having an FiO2 of 1.0. Patients on venti or face masks usually have FiO2 listed (i.e, 60% Face Mask), as do patients on High-Flow Nasal Canula, so please use listed FiO2 in that scenario.

- ◯ 0 points: PaO2/FiO2 ratio >=400, or SaO2/FiO2 ratio >301
- ◯ 1 point: PaO2/FiO2 ratio 300-399, or ratio 221-301
- ◯ 2 points: PaO2/FiO2 ratio < 300 (or SaO2/FiO2 ratio < 221)
- ◯ 3 points: PaO2/FiO2 ratio 100-199 (or ratio 67-141) AND mechanically ventilated
- ◯ 4 points: PaO2/FiO2 ratio < 100 (or SaO2/FiO2 ratio < 67) AND mechanically ventilated
- ◯ Missing

Baseline Renal SOFA Score (all baseline values during a 24-hour period before infection onset)

- ◯ 0 points: Cr < 1.2
- ◯ 1 point: Cr 1.2-1.9
- ◯ 2 points: Cr 2.0-3.4
- ◯ 3 points: Cr 3.5-4.9, or urine output < 500 cc/day
- ◯ 4 points: Cr >5.0 or urine output < 200 cc/day
- ◯ Missing

Baseline Liver SOFA Score (all baseline values during a 24-hour period before infection onset)

- ◯ 0 points: Bilirubin < 1.2
- ◯ 1 point: Bilirubin 1.2-1.9
- ◯ 2 points: Bilirubin 2.0-5.9
- ◯ 3 points: Bilirubin 6.0-11.9
- ◯ 4 points: Bilirubin>=12.0
- ◯ Missing

Baseline Platelet SOFA Score (all baseline values during a 24-hour period before infection onset)

- ◯ 0 points: Platelets >=150
- ◯ 1 point: Platelets 100-149
- ◯ 2 points: Platelets 50-99
- ◯ 3 points: Platelets 20-49
- ◯ 4 points: Platelets < 20
- ◯ Missing

Baseline SOFA score (all baseline values during a 24-hour period before infection onset) ______________________________

© 2019 Rhee C et al. *JAMA Network Open.*

Did the patient have sepsis, as defined by an acute rise in SOFA score by >=2 attributable to infection?

For patients transferred from an outside hospital, If infection does not meet sepsis/SOFA criteria at the current hospitalization, but clearly met sepsis criteria PRIOR to transfer (i.e, based on OSH notes documenting septic shock requiring pressors), please take that into account for this question.

- ◯ Definite sepsis (i.e., clear source of infection and no other obvious contributors to organ dysfunction)
- ◯ Probable sepsis (i.e.,clear or probable infection; other factors may have contributed to organ dysfunction but infection was likely the primary driver)
- ◯ Possible sepsis (i.e.,no definitive source of infection identified but patient treated for infection/sepsis and no other obvious cause of organ dysfunction, OR infection likely to be present and may have been one of several possible contributors to organ dysfunction)
- ◯ Not sepsis

If infection was not the immediate cause of death / transition to hospice, did sepsis contribute to the patient's death? Please rate on a scale of 1-6.

- ◯ 1 - Definitely contributed
- ◯ 2 - Moderately likely to have contributed
- ◯ 3 - More likely to have contributed
- ◯ 4 - Less likely to have contributed
- ◯ 5 - Moderately unlikely to have contributed
- ◯ 6 - Definitely did not contribute

Please provide rationale for how sepsis did or did not contribute to the patient's death or transition to hospice (free text)

____

SECTION V. MEDICAL CARE AND POSSIBLE ERRORS - Please only answer this section if Sepsis (Possible, Probable, or Definite) was the Cause of Death or if Sepsis was Present During Hospitalization (Possible, Probable, or Definite)

Was the patient DNR/DNI or have other limitations in care on admission? (Changes in code status or goals of care AFTER admission do not count).

- ☐ No limitations in care on admission
- ☐ DNR/DNI on admission
- ☐ Other limitations in case (e.g. patient/family did not want ICU admission, vasopressors, antibiotics, etc)

When did the patient become CMO (comfort measures only)?

- ◯ Never
- ◯ On day of admission
- ◯ On day of (or immediately proximal to) death / transfer to hospice

Date of transition to CMO (m-d-y)

____

Was there an error or suboptimal aspect to sepsis care during hospitalization? (Assuming that aggressive sepsis care was consistent with goals-of-care). Check all that apply.

For this question, with regards to patients transferred from an outside hospital, please focus primarily on the data available from your hospital.

- ☐ No errors
- ☐ Significant delay in identifying or recognizing infection/sepsis
- ☐ Delay of antibiotic administration (>3 hours) after sepsis onset
- ☐ Inappropriate antibiotic selection (bug-drug mismatch)
- ☐ Inadequate fluid resuscitation or signficant delay
- ☐ Inadequate source control or significant delay
- ☐ Other

If error in sepsis care present, please explain in more detail AND EXPLAIN WHAT COULD HAVE BEEN DONE DIFFERENTLY TO IMPROVE THE PATIENT'S OUTCOME (free text)

____

© 2019 Rhee C et al. *JAMA Network Open.*

Was there another medical error or preventable complication present during hospitalization? (Check all that apply.)

For this question, with regards to patients transferred from an outside hospital, please focus primarily on the data available from your hospital. fracture)

- ☐ No
- ☐ Denial/delay in ICU admission
- ☐ Procedure-relatedcomplication
- ☐ Medication dosing or administration error
- ☐ Unexpected adverse reactionto medicine
- ☐ Fall resulting in injury (e.g. change in consciousness, intracerebral bleed,
- ☐ Venous thromboembolism not present on admission, due to inappropriate lack of VTE
- ☐ prophylaxis Hospital-acquired infection (e.g., CLABSI, CAUTI, C.diff)
- ☐ Other

If medical error present, please explain AND EXPLAIN WHAT COULD HAVE BEEN DONE DIFFERENTLY TO IMPROVE THE PATIENT'S OUTCOME (free text)

____

SECTION VI. OVERALL ASSESSMENT OF PREVENTABILITY AND SUMMARY

Please rate the preventability of this patient's death, or transition to hospice.When considering this, take into account not just whether the medical care given was reasonable and appropriate, but optimal.

For this question, with regards to patients transferred from an outside hospital, please focus primarily on the data available from your hospital.

- ◯ 1 - Definitely preventable (e.g.,poor sepsis care or medical error that would have likely changed the outcome, and no terminal illness or condition upon arrival to the hospital)
- ◯ 2 - Moderately likely to be preventable
- ◯ 3 - Potentially preventable, under the best circumstances and optimal clinical care
- ◯ 4 - Unlikely to be preventable, even though some circumstances and clinical care may not have been optimal
- ◯ 5 - Moderately likely not to be preventable
- ◯ 6 - Definitely not preventable (e.g. due to rapidly fatal illness present on arrival to hospital)

Case Summary: Please summarize the case and why you think the death was or was not preventable (free text)

© 2019 Rhee C et al. *JAMA Network Open.*

eAppendix 2. Criteria for Definite, Probable, and Possible Sepsis

“Definite sepsis” required clear evidence of infection (i.e. positive cultures or radiography and a compatible clinical syndrome) and organ dysfunction from no discernable cause other than infection.

“Probable sepsis” required a clear or probable source of infection and organ dysfunction most likely attributable to infection but with other possible contributors present.

“Possible sepsis” included cases treated for presumed sepsis but lacked definitive evidence of infection and/or had alternative possible explanations for organ dysfunction.

The primary analysis of sepsis deaths and preventability included possible, probable, and definite sepsis cases, but a sensitivity analysis was conducted using only probable and definite sepsis cases.

Sepsis was considered to be present-on-admission if the infection leading to organ dysfunction occurred within the 48 hours of admission. Sepsis was considered to be hospital-onset if the infection leading to organ dysfunction occurred after 48 hours from admission.

© 2019 Rhee C et al. *JAMA Network Open.*
